# Dynamics of Anthropometric Indices in a Large Paired Cohort With 10 Years of Follow-Up: Paving the Way to Sarcopenic Obesity

**DOI:** 10.3389/fendo.2020.00209

**Published:** 2020-04-17

**Authors:** Maria-Dolores Santos, Miquel Buti, Carolina López-Cano, Enric Sánchez, Antonieta Vidal, Marta Hernández, Antonia Lafarga, Liliana Gutiérrez-Carrasquilla, Ferran Rius, Marta Bueno, Albert Lecube

**Affiliations:** ^1^Endocrinology and Nutrition Department, University Hospital Arnau de Vilanova de Lleida, Obesity, Diabetes and Metabolism (ODIM) Research Group, IRBLleida, University of Lleida, Lleida, Spain; ^2^Institut Català de la Salut, Unitat de Suport a la Recerca, Institut Universitari d'Investigació en Atenció Primària Jordi Gol (IDIAP Jordi Gol), Lleida, Spain; ^3^Primary Health Care Unit, Lleida, Spain; ^4^Centro de Investigación Biomédica en Red de Diabetes y Enfermedades Metabólicas Asociadas (CIBERDEM), Instituto de Salud Carlos III (ISCIII), Madrid, Spain

**Keywords:** anthropometric indices, BMI, total adiposity, lean body mass, evolution

## Abstract

**Introduction:** Paired cohort investigations assessing the evolution of anthropometric indices are scarce. Here we assessed the 10-year evolution of BMI, total body fat, and lean body mass in 50,019 participants aged 18–90 years at the time of first assessment.

**Material and Methods:** A retrospective cohort study using an electronic database that contains anonymized, longitudinal data from Primary Care medical records covering the 2007–2008 and 2017–2018 periods. Total body fat was estimated using the Clínica Universidad de Navarra-Body Adiposity Estimator formula, and the Hume formula was applied to estimate lean body mass.

**Results:** The mean BMI of participants <60 years old in the 2007–2008 period increased significantly, from 27.5 to 28.3 kg/m^2^ (*p* < 0.001). However, the BMI of older subjects decreased during the next decade, from 28.9 to 28.3 kg/m^2^ (*p* < 0.001). The estimated total body fat showed a continuous progressive increase over all ages. Finally, lean body mass showed a progressive increase until the 40s, with a plateau between 40–45 years old and an uninterrupted decrease until older ages. Also, subjects who increased their BMI by 2 kg/m^2^ during the 10-year period were mainly women and younger at baseline, with a lower initial BMI and total body fat in comparison with those who experienced a BMI decrease of ≥2.0 kg/m^2^.

**Conclusion:** The evolutions of BMI and the estimated body compositions reported here confirm that the adverse decrease in lean body mass begins in middle age. The proportion of older subjects is important when evaluating overweight and obesity prevalence in cross-sectional studies.

## Introduction

The prevalence of individuals who are overweight or obese has rapidly increased during the last three decades in many developed and developing countries (1, 2). Therefore, excess body weight has become a major public health concern worldwide, with enormous physical, social, economic, and psychological repercussions (3, 4). Demographic surveys also assume that body mass index (BMI) increases at the same rate among all subjects, with a distribution that remains constant across time. However, a rightward shift in the BMI distribution has been described, with those who are obese shifting to even higher BMI levels (5, 6). Another concern is the detection of higher waist circumference at each BMI level, further accelerating the cardiometabolic health consequences of abdominal adiposity (7). Nevertheless, the definition of obesity using BMI classification is far from perfect, as it fails to provide an accurate measure of the amount and distribution of body fat (8, 9). For this reason, when gold standard measurements, such as dual-energy X-ray absorptiometry or magnetic resonance imaging, are not available, anthropometric indices that combine traditional measurements have been developed to estimate total body fat and abdominal adiposity (10, 11). Similarly, multiple indices to estimate lean body mass have also been developed (12).

The descriptions of the rise in average BMI or prevalence of excess weight over time are mainly based on nationally representative, repeated, cross-sectional household surveys (13, 14). These studies use to collect data from different individuals drawn from the same population at successive points in time and are used for the aggregate analysis of change over time (15, 16). Therefore, these data cannot be used to detect weight changes within the same individual. On the other hand, longitudinal studies involve information directly gathered in a survey of households or individuals and follow individual persons over time. Longitudinal studies allow researchers to separate the independent effects of age, period, and cohort from trends in overweight and obesity rates (17). However, large paired cohort investigations assessing anthropometric indices between long periods are scarce. Furthermore, being able to understand who will become obese has direct implications in the quest for adequate public health interventions: for example, to determine whether high-risk individuals or the whole population should be targeted.

To delve further into anthropometric dynamics, we designed the present study to evaluate the 10-year evolution of BMI and estimated total body fat and lean body mass in the same 50,019 participants aged 18–90 years old at the time of the first assessment.

## Materials and Methods

### Ethics Approval

The study was approved by the Ethics Committee of the Primary Health Care University Research Institute (IDIAP) Jordi Gol (code 19/017-P). All patient records and information were anonymized and de-identified prior to analysis.

### Study Design and Data Source

This was a retrospective, cohort study using an electronic database that contains anonymized, longitudinal data on clinical history from Primary Care medical records. The database has information from the 29 primary care centers pertaining to the Catalan Health Institute in the province of Lleida, Spain, which serves ~400,000 subjects (80% of the total population). All data were obtained through specific software (the *Electronic Clinical station in Primary Care*; eCAP) developed by the institution and used since 2001.

Data for the first assessment was obtained covering the period between January 2007 and December 2008. Participants with missing information on height or weight, younger than 18 years and older than 90 years, and those with outlying values for BMI (BMI < 15 or BMI > 50) were excluded from our analyses. From this initial cohort, we selected those alive and with a second measurement of their BMI between January 2017 and June 2018. After these exclusions, data from 50,019 subjects who have a 10-years paired BMI were used to study longitudinal anthropometric indices dynamic. Main data of the study population is displayed in Table 1. No deaths occurred among the participants during the study period.

**Table 1:** Characteristics of the participants in the study in the 2007–2008 and 2017–2018 paired periods.

	**2007–2008**	**2017–2018**
**Total population included in the database**	**376,920**	**400,439**
**Population selected for the study**	**50,019**	**50,019**
**Women, *n* (%)**	**28,819 (57.6)**	**28,819 (57.6)**
**Age (years)**	**56.3 ± 15.7**	**66.8 ± 14.3**
**Type 2 diabetes, *n* (%)**	**1.650 (3.3)**	**3.451 (6.9)**
**Hypertension, *n* (%)**	**5.202 (10.4)**	**9.403 (18.8)**
**Pregnant women, *n* (%)**	**8,763 (2.3)**	**6,820 (1.7)**
**Subjects underwent bariatric surgery, *n* (%)**	**0 (0)**	**328 (0.6)**

The age of the individuals was stratified into the following groups: 18–29 years (only in the 2007–2008 period), 30–39, 40–49, 50–59, 60–79, 80–90, and 90–100 years (only in the 2017–2019 period). Data were also evaluated according to the definition of the elderly proposed by the United Nations, and the cut-off of 60 years was used to refer to the older or elderly persons (17).

### Assessment of Anthropometric Indices

Data for patients were extracted for each year, and the most recent values were chosen. Weight (kilograms) at each respective age and height (meters) at baseline were used to calculate anthropometric indices. Normal weight was defined as a BMI between 18.5 and 24.9 kg/m^2^, overweight when BMI was between 25.0 and 30.0 kg/m^2^, and obesity when BMI was higher than 30.0 kg/m^2^ (18). Additionally, within the obesity range, a distinction was made between type I (BMI between 30.0 and 34.9 kg/m^2^), type 2 (BMI between 35.0 and 39.9 kg/m^2^), and morbid (BMI > 40 kg/m^2^) obesity. We calculated BMI change between the 2007–2008 and 2017–2018 periods and subjects who lost ≥2.0 kg/m^2^ and those who gained ≥2.0 kg/m^2^ were identified.

Total body fat was estimated using the Clínica Universidad de Navarra - Body Adiposity Estimator (CUN-BAE) formula: −44.988 + (0.503 × age) + (10.689 × sex) + (3.172 × BMI) – (0.026 × BMI^2^) + (0.181 × BMI × sex) – (0.02 × BMI × age) – (0.005 × BMI^2^ × sex) + (0.00021 × BMI^2^ × age), where male = 0 and female = 1 for sex, and age in years (11, 18). Finally, to estimate lean body mass the Hume formula was applied: 0.32810 × weight (kg) + 0.33929 × height (cm) – 29.5336 (for males), and 0.29569 × weight (kg) + 0.41813 × height (cm) – 43.2933 (for females) (12, 19).

### Statistical Analysis

Statistical analyses were performed using the SPSS software (IBM SPSS Statistics for Windows, Version 20.0. Armonk, NY, USA). A normal distribution of the variables was established using the Kolmogorov-Smirnov test, and data are expressed as the mean ± SD, median (range), or as a percentage. A paired Student's *t*-test was used to compare the baseline data (period 2007–2008) with those obtained at the end of follow-up (period 2017–2018), while categorical variables were compared using the χ^2^ test. All “*p*” values were based on a two-sided test of statistical significance. Significance was accepted at a level of *p* < 0.05.

## Results

**Figure 1:**
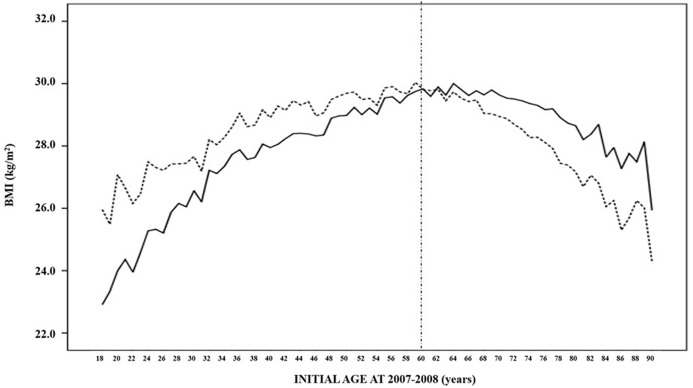
Mean BMI distribution of the 50,019 subjects in the 2007–2008 (solid line) and 2017–2018 (dashed line) paired periods according to their age at the first evaluation.

In both periods (2007–2008 and 2017–2018), the mean BMI distribution across different ages followed the same pattern: a progressive increase until the age of 60 years, and thereafter begins to diminish until older ages (Figure 1, Table 2). In addition, BMI was lower in younger than in elderly individuals, both in the 2007–2008 period [27.5 (15.0–49.9) vs. 29.0 (15.1–49.9) kg/m^2^, *p* < 0.001) and 2017–2018 period [27.7 (15.1–50.0) vs. 28.5 (15.0–57.3) kg/m^2^, *p* < 0.001] (Table 3).

**Table 2:** Evolution of mean body mass index and estimated total body fat and lean body mass by intervals of 10 years from 18 to 100 years in both periods of time (2007–2008 and 2017–2018).

	***n***	**BMI (kg/m^**2**^)**	**Estimated total body fat (%)**	***n***	**Estimated lean body mass (%)**
**18 years in 2007–2008**	**154**	**22.0 (15.5–39.0)**	**25.5 (5.6–48.6)**	**133**	**36.7 (27.5–50.2)**
**18 years in 2017–2018**	**–**	**–**	**–**	**–**	**–**
**30 years in 2007–2008**	**449**	**25.8 (16.3–45.7)**	**32.1 (11.5–54.9)**	**346**	**38.9 (28.0–59.9)**
**30 years in 2017–2018**	**194**	**26.1 (17.9–51.6)**	**33.6 (10.4–57.0)**	**168**	**38.0 (25.8–57.2)**
**40 years in 2007–2008**	**574**	**27.1 (16.2–48.4)**	**32.8 (12.2–56.4)**	**399**	**40.6 (24.9–54.4)**
**40 years in 2017–2018**	**449**	**27.0 (15.1–48.4)**	**34.6 (7.0–56.4)**	**346**	**39.5 (29.5–61.2)**
**50 years in 2007–2008**	**883**	**28.3 (15.5–49.8)**	**34.9 (13.0–57.2)**	**565**	**41.0 (25.3–55.4)**
**50 years in 2017–2018**	**574**	**28.2 (17.5–49.4)**	**35.1 (14.5–57.2)**	**399**	**40.8 (26.3–58.8)**
**60 years in 2007–2008**	**1,255**	**29.0 (18.5–49.8)**	**38.1 (17.1–56.2)**	**738**	**39.1 (26.2–58.3)**
**60 years in 2017–2018**	**883**	**29.0 (17.9–55.1)**	**36.8 (17.1–57.6)**	**565**	**41.1 (25.2–55.7)**
**70 years in 2007–2008**	**1,033**	**29.1 (15.1–49.4)**	**39.9 (20.1–57.6)**	**613**	**38.6 (25.6–58.1)**
**70 years in 2017–2018**	**1,255**	**29.0 (18.1–56.6)**	**39.1 (20.0–59.2)**	**738**	**39.2 (25.0–54.5)**
**80 year in 2007–2008**	**537**	**28.4 (18.7–49.5)**	**41.8 (21.5–57.9)**	**341**	**35.2 (20.3–50.3)**
**80 years in 2017–2018**	**1,033**	**28.4 (16.3–55.2)**	**40.1 (18.4–59.4)**	**613**	**38.1 (23.1–60.1)**
**90 years in 2007–2008**	**14**	**25.7 (20.4–31.2)**	**42.6 (27.7–47.6)**	**12**	**32.3 (28.1–39.9)**
**90 years in 2017–2018**	**537**	**26.7 (16.1–41.1)**	**41.9 (20.6–54.3)**	**341**	**34.3 (19.4–50.9)**
**100 years in 2007–2008**	**–**	**–**	**–**		**–**
**100 years in 2017–2018**	**14**	**24.3 (18.8–33.1)**	**41.6 (26.7–49.8)**	**12**	**32.0 (27.2–38.5)**

*Data are expressed as mean (range). Total body fat was estimated using the Clínica Universidad de Navarra—Body Adiposity Estimator (CUN-BAE) formula. Lean body mass was estimated using the Hume formula*.

**Table 3:** Ten-year evolution of the mean BMI and BMI categories in those participants younger and older than 60 years at the first evaluation.

			**2007–2008**	**2017–2018**	
		***n***	**Median (range)**	**Median (range)**	***p***
**Evolution of mean BMI (kg/m**^****2****^**)**					
**<60 years at 2007–2008**	**All**	**26,280**	**27.5 (15.0–49.9)**	**28.3 (15.1–58.0)**	**<0.001**
	**Men**	**11,100**	**28.5 (15.1–49.6)**	**28.7 (15.1–58.0)**	**<0.001**
	**Women**	**15,180**	**26.9 (15.0–49.9)**	**27.9 (15.2–57.3)**	**<0.001**
**≥60 years at 2007–2008**	**All**	**23,740**	**29.0 (15.1–49.9)**	**28.3 (15.0–56.8)**	**<0.001**
	**Men**	**10,101**	**28.6 (16.5–49.8)**	**28.1 (16.1–49.3)**	**<0.001**
	**Women**	**13,639**	**29.3 (15.1–49.9)**	**28.5 (15.0–56.6)**	**<0.001**
			**2007–2008**	**2017–2018**	
			**n%**	**n%**	***p***
**Evolution of BMI classification**					
<60 years at 2007–2008	Underweight		977 (3.7)	762 (2.9)	<0.001
	Normal weight		6,787 (25.8)	5,799 (22.1)	<0.001
	Overweight		9,978 (38.0)	9,711 (37.0)	<0.001
	Obesity I		5,780 (22.0)	6,406 (24.4)	<0.001
	Obesity II		2,031 (7.7)	2,552 (9.7)	<0.001
	BMI > 40		727 (2.8)	1,049 (4.0)	<0.001
≥60 years at 2007–2008	Underweight		133 (0.6)	359 (1.5)	<0.001
	Normal weight		3,254 (13.7)	4,407 (18.6)	<0.001
	Overweight		10,770 (45.4)	10,556 (44.5)	<0.001
	Obesity I		7,060 (29.7)	6,158 (25.9)	<0.001
	Obesity II		1,964 (8.3	1,780 (7.5)	<0.001
	BMI > 40		559 (2.4)	480 (2.0)	<0.001

When the effect of the 10-year evolution was assessed, the mean BMI of participants younger than 60 years in the 2007–2008 period experienced a significative increase, from 27.5 (15.6–49.9) to 28.3 (15.1–58.0) kg/m^2^ (*p* < 0.001) (Figure 1, Table 2). However, older subjects showed a reduction in their BMI during the next decade, decreasing from 28.9 (15.1–49.9) to 28.3 (15.0–55.2) kg/m^2^ (*p* < 0.001). In the same way, the 32.5% prevalence in obesity (BMI ≥ 30 kg/m^2^) in younger subjects increased to 38.1% after 10 years, whereas the percentage decreased from 40.4 to 35.9% (*p* < 0.001) among the elderly group. When a gender-specific analysis was performed, similar results were obtained (Table 3).

Different trajectories in BMI during the 10-year period were identified. Only 12.2% of the participants maintained a normal BMI throughout the entire study period. From the 10,041 participants who had a normal BMI at baseline, 3012 (30.0%) became overweight, 293 (2.9%) became class I obese, and 22 (0.2%) became class II obese, while the remaining eight achieved a BMI ≥ 40 kg/m^2^.

In addition, from the population younger than 60 years in the 2007–2008 period, we identified 15,877 participants who increased their BMI ≥ 2.0 kg/m^2^ during the 10-year follow-up, whereas 10,403 participants experienced no change or a decrease in their BMI. Subjects who increased their weight showed a higher prevalence of women (59.5 vs. 55.0%, *p* < 0.001), were younger at baseline [45.0 (18.0–59.0) vs. 48.0 (18.0–59.0) years, *p* < 0.001], and exhibited a lower initial BMI [26.9 (15.0–49.6) vs. 28.6 (15.5–49.9) kg/m^2^, *p* < 0.001], together with a lower percentage of total body fat [33.4% (3.9–57.1) vs. 35.1% (8.3–57.3), *p* < 0.001] relative to those who experienced a BMI decrease of 2.0 kg/m^2^ or more during the same period.

**Figure 2:**
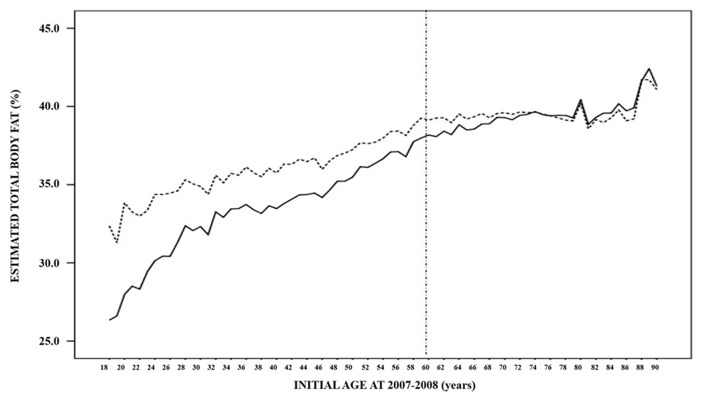
Estimated mean total body fat distribution of the 50,019 subjects in the 2007–2008 (solid line) and 2017–2018 (dashed line) paired periods according to their age at the first evlaution.

The evolution of the estimated total body fat across different ages in both periods of time (2007–2008 and 2017–2018) is represented in Figure 2. There is a continuous progressive increase, which is faster in younger than in older ages, and that achieves the highest values in the elderly. Globally, total body fat was lower in those younger than 60 years in comparison to older individuals, both in the 2007–2008 period [34.1% (3.9–57.3) vs. 39.6% (15.3–58.0); *p* < 0.001] and 2017–2018 period [36.3% (7.0–59.2) to 40.0% (16.9–59.4), *p* < 0.001] (Table 4).

**Table 4:** Ten-year evolution of the estimated mean total body fat and lean body mass in those participants younger and older than 60 years at the first evaluation.

			**2007–2008**	**2017–2018**	
		***n***	**Median (range)**	**Median (range)**	***p***
**Evolution of mean total body fat (%)**					
**<60 years at 2007–2008**	**All**	**26,280**	**34.1 (3.9–57.3)**	**36.3 (7.0–59.2)**	**<0.001**
	**Men**	**11,100**	**29.0 (3.9–49.5)**	**30.6 (7.0–53.3)**	**<0.001**
	**Women**	**15,180**	**38.7 (13.9–57.3)**	**41.2 (16.8–59.2)**	**<0.001**
**≥60 years at 2007–2008**	**All**	**23,740**	**39.6 (15.3–58.0)**	**40.0 (16.9–59.4)**	**<0.001**
	**Men**	**10,101**	**31.7 (15.3–50.3)**	**32.1 (16.9–50.2)**	**<0.001**
	**Women**	**13,639**	**44.2 (26.6–58.0)**	**44.4 (28.1–59.4)**	**<0.001**
Evolution of lean body mass (%)^*^					
**<60 years at 2007–2008**	**All**	**17,910**	**39.7 (20.1–77.8)**	**40.1 (21.5–80.7)**	**<0.001**
	**Men**	**7,506**	**44.9 (28.3–77.8)**	**45.1 (28.9–80.7)**	**<0.001**
	**Women**	**10,404**	**36.2 (20.1–55.0)**	**36.9 (21.5–53.7)**	**<0.001**
**≥60 years at 2007–2008**	**All**	**14,140**	**38.2 (20.3–73.2)**	**37.8 (19.4–79.3)**	**<0.001**
	**Men**	**6,218**	**42.8 (29.7–73.2)**	**42.5 (28.6–79.3)**	**<0.001**
	**Women**	**7,922**	**34.7 (20.3–71.5)**	**34.3 (19.4–70.2)**	**<0.001**

* *The estimation of lean body mass was only available in 30,050 subjects*.

When the effect of the 10-year evolution was assessed in those younger than 60 years, total body fat experienced a significant increase between the 2007–2008 and 2017–2018 periods [34.1% (3.9–57.3) vs. 36.3% (7.0–59.2); *p* < 0.001)]. In those aged 60 years and older, total body fat increased with a lesser intensity; from 39.6% (15.3–58.0) to 40.0% (16.9–59.4) (*p* < 0.001) (Figure 2, Table 4).

**Figure 3:**
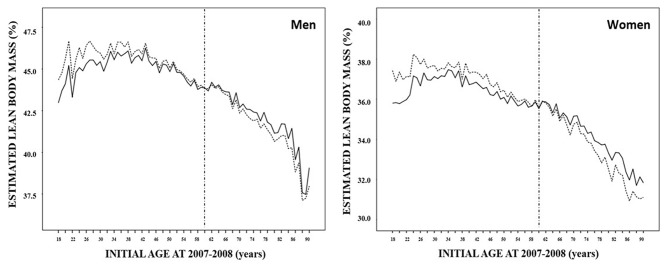
Estimated mean lean body mass distribution of 32,050 subjects in the 2007–2008 (solid line) and 2017–2018 (dashed line) paired periods according to their age at the first evaluation and sex.

Finally, estimated lean body mass showed a dissimilar trajectory compared to BMI and total body fat: from 18 years old to the 40s, lean body mass increased progressively, showed a plateau between 40 and 45 years old, and thereafter began an uninterrupted decrease until older ages (Figure 3, Table 4). Nevertheless, considering the barrier of 60 years, lean body mass was slightly higher among the younger participants in both periods of time: 39.7% (20.1–77.8) vs. 38.2% (20.3–73.2) in the 2007–2008 period, and 40.1% (21.5–80.7) vs. 37.8% (19.4–79.3) (*p* < 0.001 in both cases). When the impact of 10 years addition was assessed, lean body mass experienced a significant increase before the age of 60 years, decreasing afterward. The same dynamics were observed when data were evaluated according to gender.

## Discussion

The results of the present study describe the different evolutions of BMI and the estimated body compositions (total body fat and lean body mass) across a wide range of ages, between 18 and 90 years. Our data confirm the potential adverse decrease in lean body mass beginning in middle age, and the importance of considering the percentage of older subjects included in large cross-sectional studies evaluating overweight and obesity prevalence. In addition, the discouraging change of the three measurements (BMI, total body fat, and lean body mass) after a 10-year period is also provided.

The United Nations uses 60 years old to divide younger and older cohorts of a population (20). This is not a perfect definition of an elderly individual, as this notion has different meanings in different societies and does not accommodate influences such as chronology, transformation in social role, and change of competences. However, our data demonstrate an increase in BMI of seven units from 18 to 60 years that is followed by a transient plateau in the sixth decade of life, and a new 3.5 BMI units decrease from 70 to 90 years old. These results are also in concordance with data extracted from a systematic review and meta-analysis of epidemiological studies, carried out in 199 countries over three decades between 1980 and 2008 (21). This study showed that the average rate of BMI increase per decade in men was 0.4 and 0.5 kg/m^2^ in women (21). More interesting, when Dobson et al. analyzed seven cross-sectional health surveys conducted between 1995 and 2017–18 in Australia, prevalence of obesity increased steadily with birth age until the 1960s and then accelerated (16). In addition, the 12.6% increase in total body fat from 18 to 60 years is followed by a further 4.5% increase from 60 to 90 years old. Finally, lean body mass achieves its maximum percentage in a person's 50s and shows a global decrease of 8.7% from 50 to 90 years old. Altogether, our results pave the way for the transition from overweight to presarcopenia in the older population from Lleida, Spain (22).

The clinical definition of sarcopenia is not broadly accepted. However, there is a consensus that sarcopenia is a syndrome characterized by the progressive and generalized age-related loss of skeletal muscle mass associated with low skeletal muscle strength or low physical performance (23). Sarcopenia is associated with an increased risk of physical disability, frailty, poor quality of life, and death (24, 25). In addition, the *International Working Group on Sarcopenia* also considers that muscle mass loss might be present alone or in conjunction with increased fat mass (26). In our study, we failed to evaluate muscle function by handgrip or walking speed. However, the *European Working Group on Sarcopenia in Older People* also suggests a “presarcopenia” stage characterized only by low muscle mass without impact on muscle strength or physical performance (22). However, and more recently, the same society considers sarcopenia as a muscle disease associated with aging and older people which development begins earlier in life, with low muscle strength overtaking the role of low muscle mass as a principal determinant (27). In addition, simple measurements and specific cut-off points that identify and characterize this condition are recommended. Although we have not measured lean body mass with accurate techniques, our data indicate that the age of 60 can be used as a cut-off age to initiate measures to identify subjects more vulnerable to developing sarcopenia and to prevent or postpone, as much as possible, onset among the elderly. Similarly, our data also reinforce the importance to boost muscle in youth and young adulthood, to preserve muscle in middle age and minimize deficiency in older age (27). In fact, its prevalence is reported to be 5–13% in 60–70-year-olds, increasing to 11–50% among the population older than 80 years (25). In addition, the continuous rise of estimated total body fat might also interact with fat infiltration into muscle, contributing to decrease muscle quality and work performance (25). In this way, a bidirectional and synergistically impairment between loss of muscle mass and obesity might exist (28, 29).

Data from the cross-sectional *Study on Nutrition and Cardiovascular Risk in Spain* (ENRICA) reported a general prevalence of overweight of 39.4% as well as a prevalence of obesity of 22.9% (30). This study was carried out between June 2008 and October 2010 in 12,883 persons representative of the non-institutionalized population aged 18 years and older. Data from the well representative cohort in the province of Lleida, from 18 to 90 years old, shows that in a similar period (2007–2008), the prevalence of overweight and obesity were 41.7 and 36.2%, respectively. Differences between these studies are evident, especially in the obesity stage, and can be partially explained by the broad range of older people included in our study: whereas the ENRICA study included 2,438 subjects aged 65 years or older, the present study analyzed data from 23,740 participants between 60 and 90 years old (30). Older subjects exhibit a higher BMI than younger individuals, and BMI in the elderly is closer to obesity than to the normal weight stage. This fact also enhances the importance of including all age ranges to better estimate obesity prevalence in the general population. In this way, the cut-off of 60 years old is also important when evaluating the dynamic evolution of BMI across different periods of time. For example, the evolution of obesity prevalence between 2007–2008 and 2017–2018 in the entire population in Lleida did not change: 36.8 and 36.2%, respectively. However, the same measurement experienced a 5.6% increase among subjects aged 18–60 years old in the 2007–2008 period during the next 10 years of follow-up, whereas the prevalence of obesity decreased 4.5% in elderly individuals.

Another focus of interest remains in the ability to identify subjects at risk of gaining weight over time. More vulnerable population segments comprise children, low-income individuals, racial/ethnic minority groups, low educational level, rural populations, and adults aged >65 years (31, 32). In our 10-year paired cohort study, we have shown that preventive healthy public strategies must be focused and intensified in younger women with lower BMI. Therefore, our data support the notion that preventive strategies to enhance weight maintenance should be initiated and implemented at earlier ages.

Some potential limitations should be considered in evaluating the results of our study. First, we ignore which proportion of weight and height were self-reported by the participant or performed under standardized conditions by experienced health professionals, which makes misclassification possible, particularly among subjects with obesity. Second, other relevant information that could influence obesity prevalence and dynamics, such as socioeconomic status, educational level, dietary mistakes, behavioral gaps, or lack of physical activity, was not available in our study. We also did not exclude pregnant women or subjects who underwent bariatric surgery, despite their confounding impact on weight and waist circumference. However, our data represent a large proportion of our community, including many participants of both genders across all age frames. Third, the relationship between BMI and body composition has been estimated in our study. Direct assessment of body adiposity can be achieved using dual-energy X-ray absorptiometry or magnetic resonance imaging (33, 34). Nevertheless, the cost, complexity, and time-consuming nature of these well-established approaches, together with the retrospective analyses of the data, limit their application in this project. Fourth, because these data are gathered from medical records, they might be biased toward less healthy individuals.

In conclusion, we used data from 50,019 individuals to describe BMI distribution between the ages of 18 and 90 years, and BMI evolution during the following 10 years in the same cohort. In addition, total body fat and lean body mass were also estimated, which contributes data that helps to understand weight dynamics in the general population. Our data highlights the importance of considering the cut-off of 60 years when evaluating overweight and obesity prevalence in large cross-sectional studies. Finally, the discouraging trends in the three measurements (BMI, total body fat, and lean body mass) after a 10-year period should focus attention on reinforcing preventive strategies among younger subjects before they become overweight.

## Data Availability Statement

The datasets generated for this study are available on request to the corresponding author.

## Ethics Statement

The studies involving human participants were reviewed and approved by Primary Health Care University Research Institute (IDIAP) Jordi Gol (code 19/017-P). Written informed consent for participation was not required for this study in accordance with the national legislation and the institutional requirements.

## Author Contributions

M-DS, and MBue recruited patients, collected and analyzed data, wrote the first draft of the manuscript, and had final approval of the version for publication. CL-C and ES supervised the research, interpreted data, critically reviewed the draft of the article, and contributed to the discussion. AV, MH, ALa, LG-C, FR, MBue, and MBut collected and analyzed data, critically reviewed the draft of the article, and had final approval of the version for publication. ALe designed the study, supervised the research, analyzed and interpreted data, and wrote the manuscript.

## Conflict of Interest

The authors declare that the research was conducted in the absence of any commercial or financial relationships that could be construed as a potential conflict of interest.
